# LONELINESS AMONG ADULTS AGING WITH INTELLECTUAL AND DEVELOPMENTAL DISABILITIES: THE ROLE OF LIVING SITUATION

**DOI:** 10.1093/geroni/igae098.1655

**Published:** 2024-12-31

**Authors:** Jeffrey Stokes, Danielle Waldron, Elisabeth Stam

**Affiliations:** University of Massachusetts Boston, Boston, Massachusetts, United States; Stonehill College, North Easton, Massachusetts, United States; University of Massachusetts Boston, Boston, Massachusetts, United States

## Abstract

**Background and Objectives:**

Loneliness is a serious public health concern among the aging population. Not only is loneliness an unpleasant emotional experience, it is also associated with worse health, well-being, and even mortality. This is a particularly important issue among the population aging with intellectual and developmental disabilities (I/DD), who are more likely to experience loneliness across the life course, and who – particularly if living in an intermediate care facility (ICF) or nursing facility – may lack social connections.

**Research Design and Methods:**

We analyzed panel data from the 2013-2014 through 2021-2022 waves of the National Core Indicators In-Person Survey (NCI-IPS; 8 waves total), a national survey of adults with I/DD receiving state services (N = 101,374 observations drawn from 49 states). Multilevel logistic regression models examined whether loneliness varied according to living situation.

**Results:**

Results indicated that (1) adults aging with I/DD in ICF and nursing facilities reported significantly greater loneliness than those living in the community, and especially those living with family; (2) having friends was associated with reduced loneliness overall, yet (3) significant moderation analysis revealed that having friends was associated with reduced loneliness among those living in the community, but not for those living in ICF or nursing facilities.

**Discussion:**

These results indicate not only that adults with I/DD living in institutionalized settings are at higher risk of experiencing loneliness and its detrimental effects, but that specialized interventions are required to meet their unique needs and reduce their loneliness in mid and later life.

